# Fixed dose artesunate amodiaquine – a phase IIb, randomized comparative trial with non-fixed artesunate amodiaquine

**DOI:** 10.1186/1475-2875-13-498

**Published:** 2014-12-16

**Authors:** Bernhards Ogutu, Elizabeth Juma, Charles Obonyo, Vincent Jullien, Gwenaelle Carn, Michel Vaillant, Walter Robert John Taylor, Jean-René Kiechel

**Affiliations:** Centre for Clinical Research, Kenya Medical Research Institute, Kisumu, Kenya; Centre for Global Health Research, Kenya Medical Research Institute, Nairobi City, Kenya; INSERM U1129, University Paris Descartes, CEA Gif-sur-Yvette, France; Service de Pharmacologie, Hôpital Européen Georges Pompidou, Assistance Publique-Hôpitaux de Paris, Paris, France; Drugs for Neglected Diseases initiative, Geneva, Switzerland; Competence Centre in Methodology and Statistics, Public Research Centre for Health, Strassen, Luxembourg; Division of Tropical and Humanitarian Medicine, Geneva University Hospitals, Geneva, Switzerland

**Keywords:** Malaria, Amodiaquine, ECG, Kenya, Pharmacokinetics

## Abstract

**Background:**

Pharmacokinetic (PK) and pharmacodynamic (PD) data are limited for artesunate (AS) and amodiaquine (AQ) in uncomplicated *Plasmodium falciparum*.

**Methods:**

From 2007-8, 54 *P. falciparum*-infected, Kenyan adults were assigned randomly fixed dose (FD) ASAQ (n = 26) or non-fixed (NF) ASAQ (n = 28). Total doses were 600 mg AS (both arms) + 1,620 mg (FD) or 1,836 mg (NF)AQ. Follow-up extended over 28 days. PK data were collected for AS, dihydroartemisinin (DHA), AS + DHA combined as DHA equivalents (DHAeq), AQ, desethylamodiaquine (DAQ),and their relationships assessed against the PD collected data consisting of parasitological efficacy, adverse events (AEs), and the Bazett’s corrected QTinterval (QTcB).

**Results:**

Mean AUC 0-72 of dihydroartemisinin equivalents (DHAeq) when administered as a fixed dose (FD) compared to NF dose were similar: 24.2 ±4.6 *vs* 26.4±6.9 µmol*h/L (p = 0.68) Parasite clearance rates were also similar after 24 hrs: 17/25 (68%) *vs* 18/28(64.3%) (p = 0.86),as well as at 48 hrs: 25/8 (100%)*vs* 26 (92.9%)/28 (p = 0.49). Mean FD *vs* NF DAQ AUC_0-28_ were 27.6±3.19 *vs* 32.7±5.53 mg*h/L (p = 0.0005). Two PCR-proven new infections occurred on Day (D) 28 for estimated, *in vivo*, DAQ minimum inhibitory concentrations of 15.2 and 27.5 ng/mL. Combining the FD and NF arms, the mean QTcB at D2+4 hrs increased significantly (p = 0.0059) *vs* baseline: 420 *vs*410 ms (∆ = 9.02 (95% confidence interval 2.72-15.31 ms), explained by falling heart rates, increasing DAQ concentrations and female sex in a general linear mixed effects model. Ten of 108 (9.26%) AEs (5/arm) reported by 37/54 (68.5%) patients were possibly or probably drug related. Severe, asymptomatic neutropaenia developed in 2/47 (4.25%) patients on D28: 574/µL (*vs*D0: 5,075/µL), and 777/µL (*vs*D0: 3,778/µL).

**Conclusions:**

Tolerability of both formulations was good. For QTcB, a parameter for ECG modifications, increases were modest and due to rising DAQ concentrations and falling heart rates as malaria resolved. Rapid parasite clearance rates and no resistant infections suggest effective pharmacokinetics of both formulations.

**Electronic supplementary material:**

The online version of this article (doi:10.1186/1475-2875-13-498) contains supplementary material, which is available to authorized users.

## Background

Fixed dose (FD)artesunate-amodiaquine (ASAQ)was developed, WHO prequalified, and registered by a consortium coordinated by the Drugs for Neglected Diseases *initiative* (DND*i*)[1, 2]. Dosing is straightforward and based on four optimized, age/weight categories [3] which provides greater dosing accuracy than coblistered ASAQ [4] and good adherence [5]. As of 2014, ASAQ is available for use in 35 countries, including 32 in Africa [6]. Where tested in Africa, FD ASAQ has consistently achieved high efficacy, achieving Day 28 cure rates of ~93, 94 and 100% in children 60 months of age from Burkina Faso [6], Benin [5] and Central African Republic [10], respectively; ~98% in children <eight years of age in Cote D’Ivoire, Cameroon and Senegal [7] and <five years of age in the Democratic Republic of Congo (DRC) [8], as well as 100% in Nigerian children <12 years of age [9]. FD ASAQ was non-inferior to artemether-lumefantrine (AL) in two trials, achieving cure rates exceeding ~95% and non-inferiority margins of 3% [8, 11]. Non-fixed (NF) dose ASAQ has also achieved high cure rates across Africa [12–26] with some reports of efficacy <90% in focal parts of DRC, Tanzania, Sierra Leone, and Rwanda [26–29].

ASAQ is generally well tolerated and, across a range of studies, has a broadly similar adverse event (AE) profile as AL [5, 9, 11, 30, 31]. However, one study found significantly higher rates of fatigue, nausea, vomiting, and anaemia in the FD ASAQ arm compared to AL [32]. In this study, the relationship between Day (D)7 desethylamodiaquine (DAQ) concentrations and AE varied with age: a higher median D7 DAQ was associated with fatigue in children under the age of five, and vomiting in those aged ≥ five years of age. Rates of early vomiting or of rejecting the tablets (i.e., spitting them out) were low (0.2 to 6%) [6, 11, 32], and more common in children under five years old [DND*i* data on file [11, 32]].

In the past, AQ as malaria prophylaxis caused significant and sometimes fatal hepatotoxicity, which sometimes occurred with severe neutropaenia [33–35]; AQ is no longer used as malaria prophylaxis. Increases in liver enzymes following treatment with ASAQ or AL appear more likely in chronic hepatitis B carriers and were independent of DAQ concentrations [32]. AE rates of all grades for increased concentrations of aspartate and alanine aminotransferase (AST and ALT) ranged from 3 to 8% in both FD ASAQ and AL recipients [32]. Grade 3 (>5-20 × upper limit of normal (ULN)) and grade 4 (>×20 ULN) hepatotoxicity was rare and occurred in only 4/496 (0.8%) of patients aged > five years, not quite significantly higher (p = 0.06) than in AL-treated patients 1/502 (0.2%) [32], and in 1/529 (0.19%) of children ≤60 months of age from Burkina Faso [6]. Rates of all grade neutropaenia in patients aged > five years were 19 and 21% in 496 FD ASAQ and 502 AL recipients, respectively; corresponding rates in children < five years of age were lower: 4% (n = 149) and 5.3% (n = 150) [32] respectively. Furthermore, repeated use of ASAQ in 208 malaria-infected children from Uganda over two years (median number of treatments = 16) showed a similar AE profile to 205 children who received repeat dose AL (median number of treatments = 15). The incidence (ASAQ *vs* AL) of moderate/severe hepatic dysfunction and severe neutropaenia was low in both cohorts: five *vs* three episodes and two *vs* one episode, respectively.

Available cardiac data on AQ and ASAQ are very limited. Ngousse *et al.* found clinically insignificant prolongation of PR, QRS and the Bazett’s corrected QT interval (QTcB) in *Plasmodium falciparum*-infected adults, and no relationship between the QTcB and DAQ concentrations at 52 hours, the mean time for maximal DAQ concentrations [36]. Similarly, Adjei *et al.* failed to find a positive relationship between DAQ concentrations and either the D3 QTcB or the D3-D0 change in QTcB in African children and adolescents [37]. The lack of an apparent association between QTcB and DAQ concentrations suggest a disease effect on the QTcB but does not exclude definitively a DAQ-related cardiac effect. Intravenous AQ produced QRS interval prolongation in six out of seven healthy, Thai adult volunteers, but no significant changes in heart rate, PR or corrected QT intervals [38]. DAQ was not detected at any time, suggesting AQ caused the slowing of intracardiac conduction.

As part of the development of FD ASAQ, a phase IIb pharmacokinetic and safety study was conducted to complement population pharmacokinetic (PK) data from the phase III trial [39] and to explore PK and pharmacodynamic (PD) relationships.

## Methods

### Study design and site subject eligibility criteria

The study was an open label, phase IIb, randomized, controlled trial comparing the safety, population PKs, and efficacy of FD ASAQ and NF ASAQ. The study was conducted between November 2007 and June 2008 in the Chulaimbo Sub-District Hospital in Kisumu, western Kenya. This area has intense transmission of chloroquine-, AQ-, and sulphadoxine-pyrimethamine- resistant *P. falciparum* [40–42] as well as significant malaria-related anaemia [43]. In 2007, NF ASAQ had a polymerase chain reaction (PCR)-corrected failure rate of just under 10% in children in the same area [44]. The protocol was approved by the Kenya Medical Research Institute (KEMRI) Ethical Review Committee. This study was conducted in accordance with the principles of Good Clinical Practice and the Declaration of Helsinki. Written informed consent was obtained from patients or an independently witnessed thumb print, as appropriate.

### Inclusion/exclusion criteria

Fully informed, consenting patients aged 18 to 60 years presenting with acute, uncomplicated *P. falciparum* with an asexual parasitaemia of >1,000 parasites/µL and either a history of fever or a measured temperature of ≥37.5°C in the preceding 24 hours were enrolled. Exclusion criteria were (i) mixed *Plasmodium* infection; (ii) pregnancy (urine test for human chorionic gonadotropic hormone (Î²HCG) or lactation; (iii) clinical and/or laboratory features of severe malaria; (iv) severe malnutrition; (v) a concomitant febrile illness of any cause; (vi) significant known comorbidities e.g., cardiac, renal, hepatic diseases, HIV/AIDS; (vii) hypersensitivity to AS or AQ; (viii) an ECG abnormality requiring urgent treatment; (ix) splenectomy; and, (x) ingestion of an artemisinin in the previous three days and/or ingestion of sulphadoxine-pyrimethamine in the previous seven days.

### Randomization and treatment

Patients were computer randomized to either FD ASAQ or NF ASAQ before the trial. Treatment regimens were contained in opaque, sealed envelopes in a locked cabinet and were opened after randomization to ensure adequate concealment of drug allocation. Supervised dosing followed the manufacturer’s recommendations: (i) FD: two ASAQ tablets/day for three days; one tablet contains AS 100 mg and AQ 270 mg (*sanofi-aventis*, France; batch number 0002, expiry date June 2008) for a total dose of 600 mg AS and 1,620 mg AQ; (ii) NF: AS 200 mg/day (four tablets of 50 mg Arsumax^®^ (*sanofi-aventis*, France; batch number 060504, expiry date May 2008) + AQ 612 mg/day (four tablets 153 mg Flavoquine® (*sanofi-aventis*, France; batch number 417, expiry date July 2009) for three days, giving total doses of 600 mg AS and 1,836 mg AQ. Patients were observed for 1 hour after drug administration. A repeat dose was administered if there was early vomiting during this time. No concomitant food was given.

### Study procedures

At baseline (D0), medical and drug histories were taken, physical examinations performed, Giemsa stained thick and thin blood films were made and blood samples obtained (see below). Thick films were examined and reported as the number of parasites (N)/200 white blood cells (WBCs), converted to N/µL by assuming a total WBC count of 8,000 cells/µL. A negative film was declared when the examination of 1,000 WBCs did not reveal any asexual parasites. Basic clinical examinations were performed and malaria blood film slides prepared on D1, 2, 3, 7, 14, 21, and 28. Standard 12 lead ECGs were performed on D0 pre-dose, D0 + 2 hours, D0 + 4 hours, D2 + 2 hours, D2 + 4 hours and D28. Women underwent urine pregnancy testing at screening and on D28.

### Blood sampling

Full blood counts and liver function tests were performed on D0 (pre-dose), D7and D28, and repeated as clinically indicated. Blood samples for drug concentrations were taken from each patient at the following fixed days: D0 (pre-dose), D7, D14, D21, D28, and also randomly at the following time points: 0.25, 0.50, 1, 1.5, 2, 4 hours post-dosing on D0, and at 0.25, 0.50, 1, 1.5, 2, 4 hours on D2.

Two to three drops of blood were collected on a 3 MM filter paper (Whatman, UK) on D0, D7, D14, D21, and D28 for genotyping by PCR of three genetic markers: merozoite surface proteins (*msp1* and *msp2)* and glutamate rich protein (*glurp*). Genotyping was done for patients with recurrent infections (day of recurrence). A new infection was diagnosed if any one of the three alleles at baseline and recurrence was different; a resistant infection was diagnosed if alleles were shared at baseline and recurrence [45].

### Treatment outcomes

The WHO classification of treatment outcomes was used [46]. Early treatment failure (ETF) was defined as (i) the development of danger signs or severe malaria on D1, 2 or 3 in the presence of parasitaemia; (ii)parasitaemia on D2 greater than on D0 irrespective of oral temperature; and, (iii) parasitaemia on D3 with oral temperature ≥37.5°C; (iv) parasitaemia on D3 ≥ 25% of the count on D0. Late clinical failure (LCF) was defined as (i) development of danger signs or severe malaria after completing the full course of drug administration (D4-D28) in the presence of parasitaemia, without meeting ETF criteria previously; and, (ii) presence of parasitaemia and an oral temperature ≥37.5°C after completing the full course of drug administration (D4-D28). Late Parasitological Failure (LPF) was defined as the presence of parasitaemia from D7-D28 and an oral temperature <37.5°C without meeting ETF and LCF criteria previously. Adequate clinical and parasitological response (ACPR) was defined as the absence of parasitaemia on D28 irrespective of oral temperature and not having previously met ETF, LCF or LPF criteria.

### Rescue treatment

Patients who either failed treatment or required alternative treatment because of an AE were treated with either oral AL or intravenous quinine, as clinically indicated.

### Adverse events

An AE was defined as a new symptom, sign, clinical syndrome, or laboratory result that developed or worsened following drug administration [47] and was graded 1 to 4 (mild, moderate, severe, very severe respectively), according to the National Cancer Institute Toxicity Table [18]. Locally defined, normal laboratory ranges were used to grade laboratory AEs. AE drug relationships were determined by the physicians as unrelated, unlikely, possible, probable, and very probable. A serious AE was one of the following: (i) life-threatening event; (ii) death; (iii) hospitalization or prolongation of hospitalization; (iv) disability or incapacitation; and, (v) congenital anomaly. The following AEs were sought at each assessment: headache, weakness, anorexia, nausea, abdominal pain, itching, vomiting, diarrhoea, rhinitis, cough, vertigo, and the presence of a rash. Individual AE frequencies were modelled with a logistic model with the DAQ AUC _0–28_ (not log-transformed) as covariate. Odds Ratios and 95% confidence intervals were estimated for each AE per 1 unit increase in DAQ AUC _0–28_.

### Statistical analysis

A sample size calculation was not determined but based on a similar phase IIb study [48, 49], a sample size of 25 patients per arm was considered adequate to demonstrate the disposition of AS, dihydroartemisinin (DHA), AQ and DAQ of the two formulations and to model the rate corrected QT intervals. Treatment efficacy was determined with PCR correction of recurrent parasitaemia and excluded all patients who did not complete follow-up to D28. The safety analysis included all patients who had received at least one dose of the study drug. Data were described as frequencies (proportion), median (range) or mean (standard deviation). Proportional data were compared by Chi-squared or Fisher’s exact test, as appropriate. Wilcoxon rank sum/sign rank (skewed data) or unpaired/paired ‘t’ tests (normally distributed data) were used for continuous data (SAS v 19.1.3 software (SAS Institute, Cary, NC, USA). All tests were two-tailed and a p value of ≤0.05 was considered statistically significant.

### Pharmacokinetic methods and analysis

Plasma samples were analysed using liquid chromatography-mass spectrometry/mass spectrometry (LC-MS/MS) methods developed and validated by SYNEXEL Research International.AQ and DAQ were analysed by reversed-phase liquid chromatography (X Terra C18 MS −3.5 µm; 50 mm × 3 mm id) and MS/MS (Sciex API3000) detection in the Turbo Ion Spray positive mode. AS and DHA and the internal standard (AST-D4) were analysed by reversed-phase LC-MS/MS in the Turbo Ion Spray positive mode.

AS/DHA and AQ/DAQ concentration-time data were analysed by use of the First-Order method with Interaction of the non-linear mixed effects modelling program NONMEM (version VI, version 2.0, double precision). Population PK modelling of AQ/DAQ was reported elsewhere [50]. Concentrations below the LOQ were removed from the database. AS and DHA concentrations were fitted simultaneously, as it was the case for AQ/DAQ. Several structural pharmacokinetic models were investigated. Classical one- and two-compartment models with first order absorption were evaluated for AS and AQ. The transformations of AS into DHA and of AQ into DAQ were assumed to be linear processes. One and two compartment models were evaluated to describe the disposition of AS, DHA, AQ and DAQ. Because it was not identifiable, central distribution volumes of DHA and DAQ were fixed to 1 L. Several error models (i.e., proportional, exponential, and additive random effects model) were also investigated as means of describing interpatient and residual variabilities. Systematic testing for the influence of continuous covariates on the pharmacokinetic parameters was done by use of a generalized model, according to the following equation, by using, for example, *CL/F* and *BW*:


where *TV(CL/F)* was the typical value of the apparent clearance for a patient with the median covariate value, and *θ* was the influential factor for body weight.

The influence of binary covariate (Dosage Form, sex, ALAT) was investigated as follows:


where *θ* was equal to the influential factor for Fixed Dose Combination, Men, ALAT > 30 U/L, and was otherwise fixed to 1.

The significance of a relationship between a pharmacokinetic parameter and a covariate was assessed by use of the chi-square test of the difference between the objective functions of the basic model (without the covariate) and the model with the covariate. A covariate was retained in the model if it produced a minimum decrease in the objective function of 3.64 units (*P* = 0.05, 1 degree of freedom) and if its effect was biologically plausible. An intermediate multivariate model that included all selected covariates was then obtained. A covariate was retained in the final multivariate model if its deletion from the intermediate model led to a 6.63-point increase in the objective function (*P* = 0.01, 1 degree of freedom). At each step, the goodness of fit was evaluated by use of a graph of the weighted residuals versus time after administration of the dose (time) or versus the predicted concentrations.

The accuracy and robustness of the final population models were assessed by normalized prediction errors and visual predictive checks. The final population model parameters were used to perform 200 simulations of the database. The 2.5th and the 97.5th percentiles as well as the 50th (median) of simulated concentrations were plotted against observed concentrations.

Individual Bayesian estimates of the PK parameters were used to calculate individual areas under the curve (AUC) and elimination half-lives (T½). Individual AUC values of AS and DHA, expressed in µmol*h/L, were combined in order to obtain DHA equivalents (DHAeq).

### ECG analysis

All ECGs were read and analysed by a clinical research organization, according to a prospectively designed analytical plan. ECG intervals were measured using on-screen callipers, as previously described [49]. The QT interval was corrected using three correction formulae: (i) Bazett’s QTcB = QT/RR^0.5^; (ii) Fridericia’sQTcF = QT/RR^0.33^; and, (iii) a new correction for malaria patients: QTcN = QT/RR^0.4^ defined in the cited paper [51]. A separate paper will detail the cardiac findings. Here only the QTcB is reported. A linear mixed effects model explored the relationships between the QTcB interval over time with age, sex, treatment, temperature, AQ and DAQ concentrations, and heart rate taken as fixed factors, and a random intercept.

### Role of the sponsor

The study was funded by the DND*i* which was involved in all aspects of the study, e.g., protocol development, study oversight, data review, and intellectual contribution to this publication.

## Results

**Figure 1:**
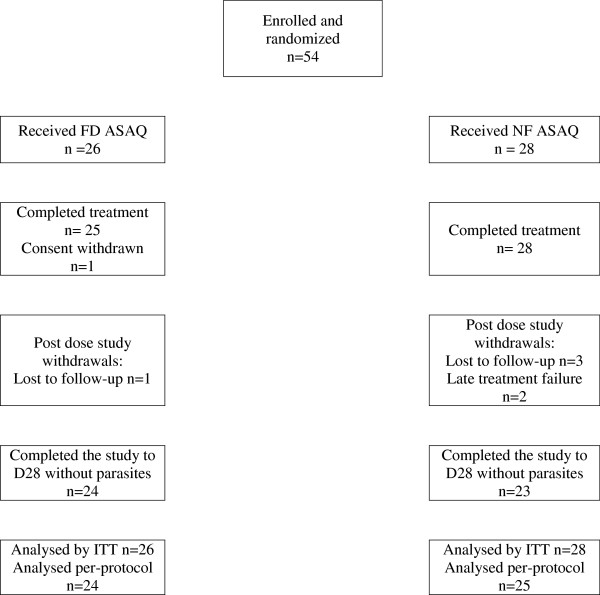
**Trial profile.**

A total of 54 patients were randomized, 26 to the FD ASAQ arm and 28 to the NF ASAQ arm. All but one patient (FD ASAQ arm) completed their treatment, four patients were lost to follow-up, and there were two late treatment failures (Figure 1). Patient baseline characteristics were similar in the two groups with the exception of the absolute neutrophil and monocyte counts (Table 1).

**Table 1:** **Patient characteristics**

Characteristic	FD ASAQ	NF ASAQ	P value
	N = 26	N = 28	
Age	23.1 (17.9-60.1)	24.2 (18.1-56.9)	0.98
Gender (n, M/F)	12/14	13/15	0.98
Luo ethnic group [N(%)]	23 (88.5)	24 (85.7)	1.00
***Symptoms*** **[N(%)]**			
Headache	23 (88.5)	26 (92.9)	0.66
Anorexia	18 (69.2)	14 (50.0)	0.15
Nausea	16 (61.5)	17 (60.7)	0.95
Weakness	13 (50.0)	15 (53.6)	0.79
Vomiting	12 (46.2)	16 (57.1)	0.42
Abdominal pain	10 (38.5)	13 (46.4)	0.55
Diarrhoea	3 (11.5)	6 (21.4)	0.47
Cough	1 (3.8)	4 (14.3)	0.35
***Signs*** **(range)**			
Weight (kg)	58.0 (50.0-90.0)	60.0 (39.0-82.0)	0.78
Temperature (°C)	37.2 (36.0-39.0)	37.2 (35.8-40.0)	0.42
Pulse rate (beats/min)	92.5 (62.0-124.0)	90.5 (57.0-131.0)	0.90
Systolic blood pressure (mmHg)	119.5 (90.0-157.0)	125.5 (85.0-160.0)	0.48
Diastolic blood pressure (mmHg)	70.0 (55.0-92.0)	74.0 (51.0-99.0)	0.37
Palpable spleen	0	1/27 (3.7)	1.0
Palpable liver	0	0	-
***Laboratory results*** **(range)**			
Asexual parasitaemia/µL	18,479 (8,927-33,915)	13,742 (5,069-23,948)	0.56
Gametocyte carriage (n, %)	1 (3.8)	0 (0.0)	0.48
Haemoglobin (g/dL)	13.3 (10.3-17.6)	13.2 (9.9-17.7)	0.53
White blood cells (× 10^3^/µL)	5.6 (5.2-6.8)	5.1 (2.3-9.5)	0.09
Neutrophils (× 10^3^/µL)	3.5 (1.3-5.6)	2.6 (0.8-8.1)	0.02
Lymphocytes (× 10^3^/µL)	1.3 (0.5-2.5)	1.3 (0.5-3.3)	0.22
Monocytes (× 10^3^/µL)	0.8 (0.6-1.1)	0.6 (0.4-0.8)	0.005
Eosinophils (× 10^3^/µL)	0.07 (0.03-0.58)	0.08 (0.05-0.19)	0.60
Platelets (× 10^3^/µL)	96.0 (44.0-229.0)	95.5 (18.0-254.0)	0.86
AST* (IU/L)	28.0 (13.4-82.9)	31.2 (15.1-56.1)	0.99
ALT* (IU/L)	21.6 (10.5-123.1)	23.2 (3.8-105.9)	0.37
Total bilirubin (µmol/L)	12.0 (1.7-50.6)	13.1 (0.0-65.3)	0.74
Creatinine (µmol/L)	70.7 (17.7-113.2)	75.6 (53.0-114.9)	0.26

All continuous data are expressed as median (range).

*AST – aspartate aminotransferase, ALT- alanine aminotransferase.

### Pharmacokinetics

The mean administered doses of AS (± SD) were similar (p = 0.56) for both arms: (i) 3.5 ± 0.11 for the FD *vs* 3.42 ± 0.008 mg/kg/d for the NF dose but were significantly lower for AQ (p = 0.001) in the FD arm: 9.24 ± 0.23 I 10.73 ± 0.34 mg/kg/d for an absolute mean and relative difference of 1.49 (95% CI 0.6-2.34) mg/kg/d and 16.1% (95% CI 6.8-25.4).

The final AQ model was a one-compartment model with first-order absorption for AQ and an irreversible transformation to DAQ; DAQ disposition was characterized by a two-compartment model. Body weight was the only significant covariate to explain the inter-individual variability of the V/F of AQ and the elimination rate constant of DAQ [50].

The mean DAQ AUC_0–28_ values were FD 27.6 ± 3.2 *vs* NF 32.7 ± 5.5 mg*h/L (p = 0.0005), a mean difference of 15.6%. This difference became non-significant (p = 0.73) when the DAQ AUC_0–28_ was divided by the total dose of AQ administered in mg: 0.051 ± 0.006 *vs* 0.053 ± 0.009 mg*h/L/mg AQ. Mean FD and NF half-lives were similar between the two arms: 204 ± 22 *vs* 214 ± 37 hours (p = 0.58), respectively.

The final AS/DHA model was a 1-compartment model with first-order absorption for AS, and first-order and irreversible transformation into DHA. Disposition of DHA was characterized by a 1-compartment model with first-order elimination.

**Figure 2:**
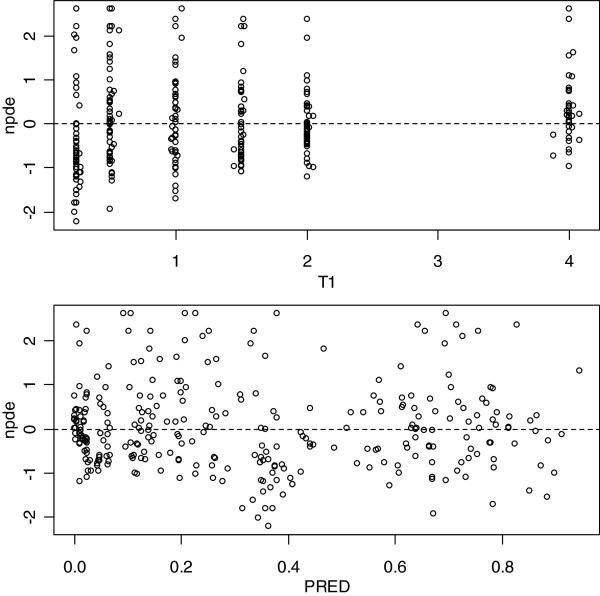
**Normalized Prediction Errors (npde) versus time after dose (T1, hours), or predicted concentrations (PRED, mg/L).**

**Figure 3:**
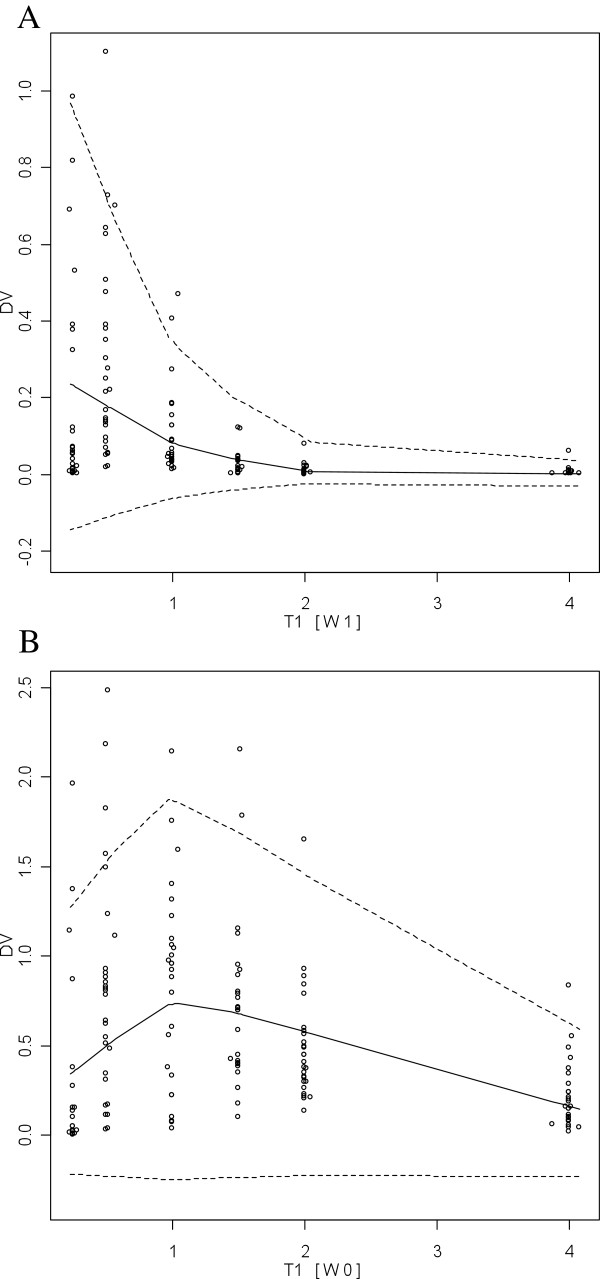
**Visual predictive checks for AS (A) and DHA (B).** DV: Dependent variable = concentration (mg/L); T1: time after dose, W1: AS, W2: DHA, circles = observed concentrations, solid line: 50^th^ percentile obtained from the simulated concentrations, lower dotted line: 2.5^th^ percentile obtained from the simulated concentrations, upper dotted line: 97.5^th^ percentile obtained from the simulated concentrations.

Body weight was found to explain the interindividual variability of the elimination rate constant of DHA (kem). However, once this covariate included, the interindividual variability of kem could not be estimated and was therefore removed from the model. Dosage form was found to influence AS bioavailability. Indeed, the bioavailability of AS was 79 % higher with the loose form. The values of the population parameters for AS/DHA for the base and final model are displayed in Table 2.Lack of bias of the Final Model was evidenced on the graphs displaying the normalized prediction errors versus population predicted concentrations or time after dose (Figure 2). Goodness of fit and lack of bias was also evidenced by the Visual Predictive Checks (Figure 3). Indeed, the AS and DHA observed concentrations were symmetrically distributed around the median of the simulated concentrations and 4.9% of the observed AS concentrations and 7.6% of the observed DHA concentrations were outside the 95% non-parametric confidence interval that was built on the simulated concentrations.

**Table 2:** **Population estimates of the base and the final model for AS/DHA**

	Base model		Final model	
Parameter	Mean	Standard error	Mean	Standard error
Ka_AS_(h^−1^)	2.45	0.34	2.41	0.34
CL/F_AS_ (L/h)	797	102	616	98.4
V/F_AS_(L)	119	51.8	85.2	36.0
FM(%)	0.81	0.16	0.00781	0.00108
Kem (h^−1^)	0.819	0.266	0.742	0.166
ѲBW_kem_	NA	NA	1.65	0.46
ѲFORM	NA	NA	1.79	0.36
ω^2^CL/F_AS_	0.101	0.089	NE	NE
ω^2^V/F_AS_	1.94	2.23	2.21	2.27
ω^2^kem_DHA_	0.000352	0.0666	NE	NE
σ^2^ad_AS_	0.000218	0.000122	0.000277	0.00015
σ^2^mult_AS_	0.665	0.154	0.674	0.116
σ^2^ad_DHA_	0.0438	0.0299	0.0324	0.0223
σ^2^mult_DHA_	0.391	0.093	0.374	0.0726

Ka_AS_: absorption rate constant of AS, CL/F_AS_: apparent clearance of AS, V/F_AS_: apparent distribution volume of AS, FM: fraction of AS metabolized into DHA, K_34_ and K_43_: distribution rate constants of DAQ, Kem: elimination rate constant of DHA, ѲBW_km_: influential factor of BW on DHA kem, ѲFORM : influence of dosage form on AS bioavailability, ω^2^CL/F_AS_: interindividual variability of CL/F_AS_, ω^2^V/F_AS_: interindividual variability of V/F_AS_, σ^2^mult_AS_: proportional residual error for AS, σ^2^ad_AS_: additive residual error for AS , σ^2^mult_DHA_: proportional residual error for DHA, σ^2^ad_DHA_: additive residual error for DHA, NA: Not Available, NE: Not Estimated.

The AUC_0–72_ values as DHAeq were similar (mean and standard deviation) between the two forms: 24.2 ± 4.6 (FD) and 26.4 ± 6.9 (NF) µmol.h/L (p = 0.68).

### Adverse events

Over 28 days, a total of 37/54 (68.5%) patients reported a total of 108 AEs: 18/26 (69.2%) FD *vs* 19/28 (67.9%) NF (p = 0.9). Ten AEs (five/arm:19.2% FD, 17.9% NF) were considered to be possibly or probably related to ASAQ treatment (Table 3). Early vomiting requiring redosing occurred in one (3.8%) FD recipient and another FD-treated patient developed an aphtous ulcer, but was not neutropaenic during the study. Absolute neutrophil counts ranged from 2,618 to 3,526 neutrophils/µL blood.

**Table 3:** **Summary of the solicited treatment emergent adverse events (TEAEs) reported at least once by 37 patients**

	TEAEs (% of patients)			Possibly or probably drug-related TEAEs (% of patients)		
Description	FD ASAQ	NF ASAQ	All	FD ASAQ	NF ASAQ	All
	(N = 26)	(N = 28)	(N = 54)	(N = 26)	(N = 28)	(N = 54)
At least one TEAE	18 (69.2)	19 (67.9)	37 (68.5)	5 (19.2)	5 (17.9)	10 (18.5)
Headache	10 (38.5)	12 (42.9)	22 (40.7)	-	-	-
Weakness	8 (30.8)	11 (39.3)	19 (35.2)	-	2 (7.1)	2 (3.7)
Anorexia	7 (26.9)	7 (25.0)	14 (25.9)	1 (3.8)	-	1 (1.9)
Nausea	6 (23.1)	8 (28.6)	14 (25.9)	-	-	-
Abdominal pain	6 (23.1)	7 (25.0)	13 (24.1)	1 (3.8)	1 (3.6)	2 (3.7)
Itching	6 (23.1)	3 (10.7)	9 (16.7)	3 (11.5)	2 (7.1)	5 (9.3)
Vomiting	4 (15.4)	2 (7.1)	6 (11.1)	-	-	-
Diarrhoea	2 (7.7)	2 (7.1)	4 (7.4)	-	1 (3.6)	1 (1.9)
Rhinitis	2 (7.7)	2 (7.1)	4 (7.4)	1 (3.8)	-	1 (1.9)
Cough	1 (3.8)	2 (7.1)	3 (5.6)	-	-	-

All FD *vs* NF ASAQ comparisons were not statistically significant.

There was no relationship between the DAQ AUC_0–28_ and the odds of reporting headache, weakness, anorexia, nausea, vomiting, abdominal pain, or itching (Additional file 1).

One NF-treated female had two serious AEs. She was admitted to hospital for symptomatic recurrent *P. falciparum* malaria (40,365 parasites/µL) on D28 and was treated with AL. She was also thought to have typhoid fever and was treated empirically with oral ciprofloxacin. No deaths were reported.

### Biochemical and haematological parameters

Because the differences in the haematological and biochemical parameters between the arms were not statistically different, they have been combined (Table 4). Over time, the mean haemoglobin (Hb) concentration nadired on D7, rising thereafter, but was significantly lower on D28 vs. baseline. The mean neutrophil and monocyte counts declined significantly over time whilst the mean lymphocyte and eosinophil counts increased significantly. One patient (NF) had severe neutropaenia of 795 neutrophils/µL at baseline, which rose to 1,200 neutrophils/µL by D28. Two out of 47 (4.25% (95% CI: 0.5 to 14.5%)) patients developed severe (grade 3) neutropaenia on D28: (i) FD: 574/µL (D0: 5,075/µL), and (ii) NF: 777/µL (D0: 3,778/µL). Both patients were well and afebrile. Six patients had severe D0 thrombocytopenia (platelets <50×10^3^/µL) which returned to normal by D7 or D28. Of the biochemical values, only the mean total bilirubin declined significantly over time. There were no abnormal AST, ALT or creatinine values throughout the study.

**Table 4:** **Summary of mean (standard deviation) and mean changes in haematological and biochemical data**

	D0	D7	D28	∆	p	∆	p
				D7-D0	D7-D0	D28-D0	D28-D0
***Haematology***							
Haemoglobin (g/dL)	13.4 (2.1)	12.0 (1.8)	12.6 (1.8)	−1.4	<0.000	−0.8	<0.000
White blood cells*	5.4 (1.4)	6.3 (1.9)	5.6 (2.3)	0.9	0.0012	0.3	0.44
Neutrophils*	3.1 (1.4)	2.6 (1.1)	2.2 (1.5)	−0.4	0.046	−0.9	0.00046
Lymphocytes*	1.3 (0.6)	2.6 (0.9)	2.4 (0.9)	1.2	<0.000	1.0	<0.0000
Monocytes*	0.71 (0.28)	0.66 (0.26)	0.48 (0.24)	−0.05	0.25	−0.22	<0.000
Eosinophils*	0.13 (0.16)	0.37 (0.43)	0.41 (0.47)	0.24	<0.000	0.28	<0.0000
Platelets*^†^	96.0 (18–254)	234 (187–311)	175 (140–213)	126.0	<0.000	81.0	<0.0000
***Biochemistry***							
AST^¶^ (U/L)	32.9 (13.2)	33.0 (18.7)	28.3 (11.0)	1.1	0.68	−4.4	0.0268
ALT^¶^ (U/L)	29.5 (21.8)	33.8 (20.8)	22.0 (10.2)	5.7	0.03	−8.1	0.005
Total bilirubin^‡^	16.9 (14.8)	7.2 (7.9)	7.0 (6.6)	−10.0	<0.000	−9.4	0.00014
Creatinine^‡^	73.5 (20.3)	71.8 (17.6)	71.9 (27.4)	−2.3	0.50	−3.4	0.48

*x 10^3^/µL; ^‡^µmol/L; ^†^median (range); ^¶^AST – aspartate aminotransferase; ALT- alanine aminotransferase.

### ECG data

A total of 363 ECGs recorded were analysed. Because there were no statistically significant differences in the mean QTcB, median PR and QRS intervals, and mean/median changes between the two arms at any time point, the QTcB data were combined for analysis. QRS intervals remained within the normal range at all time points (Table 5). At D2 + 4 hours, the mean increase in the QTcB over baseline was 9.02 milliseconds (ms) (95% confidence intervals: 2.72-15.31 ms (p = 0.0059). The nonlinear mixed effects modelling identified heart rate (inverse relationship), DAQ concentrations (positive relationship) and female sex (positive relationship) as significant, independent, explanatory variables for the QTcB over time (Table 6).

**Table 5:** Median (interquartile range) and median changes from baseline of the Bazett’s corrected QT interval, heart rate, and PR and QRS intervals for both artesunate amodiaquine arms combined*

Statistical parameter	Median	Lower quartile	Upper quartile	∆ ***vs***D0	p ***vs***D0	Median	Lower quartile	Upper quartile	∆ ***vs***D0	p ***vs***D0
Variable	QTcB interval ms					Heart rate b/min				
D0, pre-dose	410	394	425			92	80	103		
D0 + 2 h	412	398	428	2	0.3652	85	74	98	−5	0.0012
D0 + 4 h	416.5	397	429	3.5	0.2593	79	71	89	−8.5	<.0001
D2, pre-dose	411	388	423	−4.5	0.2551	59	53	64	−35	<.0001
D2 + 2 h	415	393	433.5	3	0.4897	59.5	54	64.5	−36	<.0001
D2 + 4 h	420	400	431	7.5^†^	0.0059	59	55	65	−31.5	<.0001
D28	398	384	418	−10.5	0.0016	66	59	74	−25	<.0001
Variable	QRS interval ms					PR interval ms				
D0, pre-dose	78	72	88			151.5	140.5	172.5		
D0 + 2 h	82	72	89	2	0.2572	158	146	176	5	0.0061
D0 + 4 h	82	76	88	1.5	0.1122	161	153	175	10	<.0001
D2, pre-dose	84	74	96	2.5	0.0084	163	154	185	12	<.0001
D2 + 2 h	85	79	95.5	6	0.0001	167	155.5	184.5	13.5	<.0001
D2 + 4 h	86	78	95	5	0.0003	172	151	187	15	<.0001
D28	86	76	92	5.5	0.0073	164	147	179	12	<.0001

*Only the QTcB data are normally distributed but the median interquartile ranges are shown for all parameters for clarity.

^†^Corresponding mean 95% confidence intervals are: 9.02 (2.72-15.31) ms.

**Table 6:** **Final random effects model summarizing the significant, independent factors to explain the changes in the QTcB interval over time**

Effect	Sex	Estimate	Standard error	DF	t Value	Pr > |t|
Intercept		373.06	9.3613	52	39.85	<0.0001
Time		−0.016	0.00421	296	−3.8	0.0002
Age		0.4153	0.215	296	1.93	0.0543
Sex	F	20.8761	4.6453	296	4.49	<0.0001
Sex	M	0	.	.	.	.
ECG heart rate		0.1461	0.06916	296	2.11	0.0355
DAQ concentrations		0.02972	0.01026	296	2.9	0.0041

### Efficacy

There were no ETFs in either arm. Two NF ASAQ-treated female patients developed symptomatic, new infections on D28 for PCR-corrected ACPR rates of 24/24 FD and 23/23 NF (p = 0.49) patients. Pertinent clinical and PK data for the two new infections were: (i) Patient A: D0 parasitaemia: 16,815 parasites/µL, D28 parasitaemia: 40,365 parasites/µL, weight 53 kg, AS dose: 3.7 mg/kg/d, AQ dose: 11.5 mg/kg/d, estimated DAQ AUC_0–28_ 33.23 mg*h/L, estimated DAQ concentrations were 27.5 ng/mL (D21) and 11.9 ng/mL (D28), and estimated DAQ T½ was 212 hours (8.3 days); (ii) Patient B: D0 parasitaemia: 20,860 parasites/µL, D28 parasitaemia: 19,228 parasites/µL, weight 44 kg, AS dose: 4.54 mg/kg/d, AQ dose:13.9 mg/kg/d, estimated AUC_0–28_ 32.77 mg*h/L, estimated DAQ concentrations were 15.3 ng/mL (D21) and 9.31 ng/mL (D28), and estimated DAQ T½ 214 hours (8.9 days).

On D1, persistent parasitaemia was present in 8/25 (32%) FD and 10/28 (35.7%) NF (p = 0.86) patients. No FD patient had parasites on D2compared to 2/28 (7.1%) for the NF (p = 0.49) patients. Both patients were aparasitaemic on D3. One patient in the FD and none in the NF arm had D0 gametocytes. From D2 to D28, only one FD patient had gametocytes on D14.

## Discussion

In this small group of semi-immune Kenyan adults, both ASAQ formulations were well tolerated, produced modest changes in the ECG and were effective for treating acute, uncomplicated, *P. falciparum*. Two patients developed asymptomatic severe neutropaenia on D28.

The AE profile of both ASAQ formulations was similar and clinically reported AEs were unrelated to DAQ exposure; however, there was a trend in the reporting of fatigue and increased DAQ exposure. In their large study, Shramm *et al.* found a positive relationship between D7 DAQ and the reporting of fatigue in children < five years of age. Itching was reported by ~17% of patients, a higher rate compared to the <1-3% reported in paediatric studies [5, 9, 11, 30], and may be related to adults being better able to express themselves. No patients needed parenteral rescue treatment for persistent drug-induced vomiting and only one patient needed to be redosed. These tolerability data appear more satisfactory than those from young children in whom ~7.5% required redosing [31] and ~2% required parenteral rescue treatment for persistent drug-induced vomiting [6, 30].

Asymptomatic, severe neutropaenia was found in two patients (~4%) on D28, both of whom had normal neutrophil counts at baseline. Data on the natural dynamics of neutrophils show that when malaria patients of all ages are treated with a variety of anti-malarial drugs, there is a natural decline in the mean neutrophil count whilst lymphocytes increase post-treatment [52]. Rates of post-treatment neutropaenia of all grades were similar between ASAQ (13%), other ACT (14%) and monotherapy (15%), and the risk of developing neutropaenia was inversely related to age. The rate of severe (grade 3/4) neutropaenia for all drugs combined was 3% with no significant difference between ASAQ and other anti-malarial drugs [52]. These data, therefore, do not suggest ASAQ recipients are at a higher risk of developing neutropaenia of any grade compared to other antimalarial drugs. Adjei *et al.* demonstrate an almost identical decline in the mean neutrophil counts over 28 days between NF ASAQ and AL in malaria patients aged up to 14 years [30]. Post- treatment severe neutropaenia is usually asymptomatic or mildly symptomatic and can last for up to four weeks [11, 30].

There was a small but statistically significant increase in the mean QTcB interval of ~9 ms (upper 95%, CI 15 ms), four hours after the last dose of ASAQ, representing a maximum increase of <4% over baseline. This time point corresponds to the mean maximum DAQ concentration and when malaria is resolved/resolving. Indeed, increasing DAQ concentrations and decreasing heart rate were significant explanatory factors along with female sex. The positive relationship between DAQ and QTcB contrasts with the findings of Ngousse *et al.* and Adjei *et al*. who analysed their data by simple correlation - an analysis that may have lacked sensitivity[36, 37]. The QTcB increases observed in the treated patients are consistent with other malaria studies [49, 53–56] and suggest FD ASAQ is cardiac safe at therapeutic doses.

The AS, DHA, AQ, and DAQ population PK-modelled data [50] were consistent with other published studies [39, 57–59]. AS bioavailability when administered as a FD was less compared to the NF [50] but DHA_eq_s were similar. Interestingly, this is consistent with FD AS mefloquine in Thai adults [48] but contrasts with children aged <60 months from Burkina Faso who had similar AS bio-availabilities with the same FD and NF ASAQ formulations used in this study [39]. AS is essentially a prodrug for DHA and both share similar parasiticidal properties. DHA_eq_ is probably a better PK marker, so a reduction in the bioavailability of AS exposure *per se* is of little clinical importance in the initial parasiticidal effect, as shown in the patients who had rapid falls in parasite counts in both arms, consistent with ASAQ-treated children from Burkina Faso [6]. The mean administered AQ dose and consequently the DAQ AUC_0–28_ were significantly lower (~16% lower) in the FD arm compared to the NF arm. FD ASAQ was designed for dosing simplicity across four age/weight bands. The good efficacy record of FD ASAQ across most of Africa supports the use of this simplified dosing regimen [5–7, 9, 11].

The NF arm had two, symptomatic, new infections on D28, representing a failure of post-treatment prophylaxis in an area of intense transmission of CQ and AQ resistant malaria [44]. *P. falciparum* parasites become visible by light microscopy after an interval of at least six days following sporozoite innoculation. The *in vivo* minimum inhibitory concentrations (MICs) of DAQ for the falciparum parasites in these two patients approximates the D21 DAQ concentrations, estimated at ~15 and ~28 ng/mL. These values are equivalent to ~45 and ~85 nmol/mL and are below and above one definition (≥60 nmol/mL) of *in vitro* DAQ resistance [60], suggesting at least one new infection could be DAQ resistant.

Phase IIb studies involve low numbers of patients which limits the ability to detect small differences of given parameters. Nevertheless, the distinct properties in the PK characteristics of the two formulations studied and the key factors affecting the modest increase in QTcB at the time of maximum DAQ concentrations were demonstrated.

## Conclusion

This phase II study in adults has demonstrated the cardiac safety of ASAQ at therapeutic doses and similar PK characteristics of AS and DHA. FD ASAQ is a good option where AQ resistance is low or where national malaria control programmes wish to switch from efficacious NF ASAQ to FD ASAQ.

## Electronic supplementary material

Additional file 1: **Odds ratio estimates, with Wald 95% confidence intervals, for reporting certain symptoms as adverse events per one unit increase in the desethyl amodiaquine AUC**_**0–28**_ **.** (DOCX 14 KB)
