# Supplementary material for: Fixed dose artesunate amodiaquine – a phase IIb, randomized comparative trial with non-fixed artesunate amodiaquine
Source: Malar J. 2014 Dec 16;13:498. doi: 10.1186/1475-2875-13-498 (PMC4302156; doi:10.1186/1475-2875-13-498)
Supplement: Supplementary file 1 — Additional file 1: Odds ratio estimates, with Wald 95% confidence intervals, for reporting certain symptoms as adverse events per one unit increase in the desethyl amodiaquine AUC 0–28 . (DOCX 14 KB) [file 12936_2014_3660_MOESM1_ESM.docx]

Additional file 1. Odds ratio estimates, with Wald 95% confidence intervals, for reporting certain symptoms as adverse events per one unit increase in the desethyl amodiaquine AUC_0-28._

| **Effect** | **Point Estimate** | **95% confidence intervals** | **P value** |
| --- | --- | --- | --- |
| Headache | 0.969 | 0.872-1.078 | 0.5625 |
| Weakness | 0.889 | 0.787-1.004 | 0.059 |
| Anorexia | 0.956 | 0.853-1.072 | 0.4425 |
| Nausea | 0.971 | 0.866-1.089 | 0.6135 |
| Abdominal Pain | 1.025 | 0.902-1.163 | 0.7092 |
| Itching | 1.087 | 0.917-1.289 | 0.3375 |
| Vomiting | 1.08 | 0.884-1.32 | 0.4508 |
